# ASO Author Reflections: Risk Stratification of Sentinel Node Metastases in Stage III Melanoma Patients in the Era of Adjuvant Systemic Therapy

**DOI:** 10.1245/s10434-022-12847-9

**Published:** 2022-12-20

**Authors:** Zahra Hussain, Marc Moncrieff

**Affiliations:** 1 Department of Plastic and Reconstructive Surgery, Norwich University Hospital, Norwich, UK; 2 Norwich Medical School, University of East Anglia, Norwich, UK

## Past

Before and after the publication of the Multicenter Selective Lymphadenectomy Trial 1 (MSLT-1) study data,^1^ much impassioned debate ensued as to the potential therapeutic benefit of sentinel node biopsy (SNB) for a subgroup of patients with intermediate-thickness melanoma (Breslow thickness 1.2–3.5 mm). With the advent of adjuvant systemic immunotherapy (ASI) for resected stage III melanoma, this aspect of SNB has largely been overlooked because the indications for systemic treatment have relentlessly broadened to include virtually everyone^2^ regardless of either the micrometastatic tumor burden in the SNB or the original eligibility criteria for the phase III trials that heralded the dramatic sea change in the management of resected stage III disease in the first place.

## Present

This work by Hussain et al.^3^ is an extension of the research recently undertaken by an international consortium, which stratified the risk recurrence and death in a pT1b-pT2a SNB-positive (American Joint Committee on Cancer [AJCC] IIIA) cohort of patients.^4^ In that study, maximum tumor deposit size (MTDS) was found to be the optimal phenotypical biomarker for this ostensibly low-risk group. The latest analysis of the current authors found that MTDS also was an important stratifying phenotypical biomarker for SNB-positive pT2b-pT4a primary melanomas, and that value was constant at 0.7 mm. Those “low-risk” patients with an MTDS of 0.7 mm or smaller had a prognosis remarkably similar to that of the patients classified as N0 (SNB-negative), and the authors hypothesize that it is this subgroup which may have derived therapeutic benefit from having that small metastatic focus resected at the time of their SNB. Of particular note, approximately 50 % of the SNB-positive patients at low risk by tumor burden are classified as AJCC IIIB or IIIC (8th edition) and would be offered ASI according to current clinical guidelines. The authors were unable to identify any low-risk pT4b patients, in line with the original MSLT-1 study report.^1^

## Future

If the data from this study are validated elsewhere, the results are signaling that the indications for ASI as treatment of micrometastatic stage III melanoma need an urgent reappraisal. Most current international guidelines recommend treatment based on the inappropriate application of the current AJCC classification system, which conflates both micrometastatic and macroscopic disease in the same prognostic subgroups.^5^ The key phenotypical risk factors for staging primary melanoma are microscopic tumor burden (Breslow thickness) and tumor ulceration.^5^ The data of this study suggest that similar phenotypical biomarkers, namely MTDS and extracapsular spread,^6^ can likewise stratify micrometastatic nodal disease for intermediate-thickness melanomas (AJCC pT1b-pT4a) and guide the indications for ASI accordingly. Given the poor prognosis of pT4b tumors, regardless of SNB status, and the recent approval of ASI for AJCC IIC disease, the therapeutic utility of staging this specific subgroup with SNB also needs re-evaluation.

## References

[1] Morton DL, Thompson JF, Cochran AJ, Mozzillo N, Nieweg OE, Roses DF (2014). Final trial report of sentinel node biopsy versus nodal observation in melanoma. N Engl J Med..

[2] NCCN Melanoma Guidelines Version 3.2022 [Internet]. NCCN. 2022 [cited 2022 Sep 12]. Available from: https://www.nccn.org/guidelines/guidelines-detail?category=1&id=1492.

[3] Hussain Z, Heaton MJ, Snelling AP (2022). Risk stratification of sentinel node metastasis disease burden and phenotype in stage III melanoma patients. Ann Surg Oncol.

[4] Moncrieff MD, Lo SN, Scolyer RA, Heaton MJ, Nobes JP, Snelling AP (2022). Clinical outcomes and risk stratification of early-stage melanoma micrometastases from an international multicenter study: implications for the management of American Joint Committee on Cancer IIIA disease. J Clin Oncol.

[5] Gershenwald JE, Scolyer RA, Hess KR, Sondak VK, Long GV, Ross MI (2017). Melanoma staging: evidence-based changes in the American Joint Committee on Cancer eighth-edition cancer staging manual. CA Cancer J Clin..

[6] Lo M, Robinson A, Wade R, Peach H, Dewar D, Heaton M (2021). Extracapsular spread in melanoma lymphadenopathy: prognostic implications, classification, and management. Ann Surg Oncol..

